# Nutritional Status, Body Composition, and Frailty in Community-Dwelling and Institutionalized Albanian Older Adults: A Cross-Sectional Study

**DOI:** 10.3390/nu18091379

**Published:** 2026-04-28

**Authors:** Sadmira Gjergji, Stefania Moramarco, Angela Andreoli, Fabian Cenko, Ersilia Buonomo, Alketa Bicja, Leonardo Palombi

**Affiliations:** 1 Faculty of Medicine, Catholic University of Our Lady of Good Counsel, 1026 Tirana, Albania; s.gjergji@unizkm.al (S.G.); f.cenko@unizkm.al (F.C.); ersilia.buonomo@uniroma2.it (E.B.); alketa.bicja@yahoo.com (A.B.); leonardo.palombi@gmail.com (L.P.); 2 PhD School of Nursing and Public Health, University of Rome Tor Vergata, 00133 Rome, Italy; 3 Department of Biomedicine and Prevention, University of Rome Tor Vergata, 00133 Rome, Italy; stefania.moramarco@uniroma2.it; 4 Department of Systems Medicine, University of Rome Tor Vergata, 00133 Rome, Italy

**Keywords:** older adults, Albania, frailty, nutritional assessment, body composition, Mini Nutritional Assessment (MNA), bioelectrical impedance analysis (BIA), body cell mass index (BCMI)

## Abstract

**Background**: Albania has undergone a rapid demographic transition characterized by pronounced population aging. Comprehensive geriatric assessment—functional performance, validated nutritional screening tools, and systematic evaluation of morbidities—is essential for accurately characterizing frailty and identifying the risk of malnutrition in its early stages. The objective of the present study was to improve the assessment of the health status of Albanian older adults, both community-dwelling and residing in long-term care facilities, by addressing both functional and nutritional components. **Methods**: This observational study included Albanian older adults aged ≥ 65 years, both institutionalized and community-dwelling. Frailty and nutritional status were assessed using validated questionnaires (Grauer Geriatric Functional Evaluation and Mini Nutritional Assessment—MNA), alongside body composition analysis performed by bioelectrical impedance analysis (BIA). **Results**: Data for 123 older adults were analyzed (56.9% female; mean age 71.3 ± 7.4 years; 54.5% institutionalized vs. 45.5% community-dwelling). A high prevalence of frailty and multimorbidity was observed, particularly among institutionalized older adults. With regard to nutritional status, marked age-related differences were identified among females, with a pronounced deterioration in those aged over 75 years. Body-composition-derived parameters identified a substantially higher proportion of individuals at risk of malnutrition compared with other conventional anthropometric measures. Low body cell mass index (BCMI) and institutionalization were the factors with the strongest independent associations with frailty (AOR 5.02, 95% CI 1.69–14.87, *p* = 0.004, and AOR 5.71, 95% CI 1.76–18.54, *p* = 0.004, respectively), while low BCMI was the only variable associated with an increased risk of malnutrition (AOR 4.88, 95% CI 1.78–13.40, *p* = 0.002). **Conclusions**: These exploratory findings suggest that incorporating body composition parameters into geriatric assessment may provide complementary information alongside traditional screening tools to support the development of targeted preventive and therapeutic strategies.

## 1. Introduction

Over the past three decades, Albania has undergone a rapid demographic transition characterized by pronounced population aging occurring over a relatively short period, primarily as a consequence of declining fertility and mortality rates. According to data from the 2023 Albanian Population Census, individuals aged 65 years and older account for 19.5% of the total population, representing a substantial increase compared with 11.3% in 2011 and 7.5% in 2001 [1].

In addition to demographic aging, sustained and widespread emigration has profoundly reshaped Albania’s population structure. Since the early 1990s, Albania has recorded one of the highest migration rates relative to population size in Central and Eastern Europe. This large-scale emigration has generated significant social consequences, particularly for older adults. Many elderly individuals have been left behind by emigrating family members, contributing to the emergence of socially isolated “elderly orphans,” a phenomenon that is especially prevalent in rural areas [2].

According to other authors, older people in Albania face numerous socioeconomic challenges, along with increasing levels of loneliness—similar to trends observed in other Eastern European countries—which pose imminent challenges for the support and care of the elderly [3,4].

As is well known, aging is a complex biological process involving progressive physical and psychological changes that, in interaction with environmental and social determinants, may accelerate or decelerate functional decline. In the context of Albania’s rapidly aging population, a corresponding increase in the prevalence of frailty is therefore expected. Frailty is a clinically identifiable condition characterized by diminished physiological reserve and increased vulnerability to adverse health outcomes, resulting from the cumulative decline of multiple physiological systems associated with aging [5,6]. It is increasingly recognized as a multidimensional condition encompassing nutritional, functional, cognitive, and clinical domains. Frailty frequently coexists with multimorbidity, and the presence of multiple chronic conditions significantly accelerates functional decline in older adults.

Nutritional status represents a key determinant of frailty, particularly in older adults affected by multiple chronic diseases [7]. It is closely linked to health outcomes and functional capacity in older adults. Age-related physiological changes—especially the progressive loss of metabolically active lean tissue—may impair appetite, digestion, and nutrient absorption, thereby increasing the risk of inadequate dietary intake and unfavorable changes in body composition, which are highly prevalent and strongly associated with reduced functional capacity [8]. In geriatric populations, particularly among institutionalized individuals, malnutrition is common and often manifests as involuntary weight loss, reduced autonomy, and an increased incidence of clinical complications [9]. These conditions substantially compromise quality of life and are associated with increased morbidity and mortality.

Institutionalization represents an additional and important determinant of frailty risk. Older adults residing in long-term care facilities often present with a higher burden of morbidities, lower levels of physical activity, and a greater prevalence of malnutrition compared with community-dwelling peers, thereby accelerating unfavorable changes in body composition and functional decline [10,11]. In Albania, the number of older individuals potentially requiring long-term care is increasing; however, the healthcare system faces significant challenges, and the long-term care sector remains underdeveloped, with notable structural and organizational limitations [12,13].

Comprehensive geriatric assessment, integrating functional performance measures, validated nutritional screening tools, and systematic evaluation of morbidities, allows for a more accurate characterization of frailty and its underlying determinants [14]. The Mini Nutritional Assessment (MNA), a validated multidimensional tool specifically designed for older adults, is used to assess nutritional status by integrating dietary intake, anthropometric measurements, and functional indicators to identify malnutrition and nutritional risk. Bioelectrical impedance analysis (BIA) provides non-invasive estimates of body composition, including fat-free mass, hydration status, and body cell mass. In this context, body composition screening and monitoring in older adults have been strongly recommended to prevent malnutrition and related disorders [15]. In fact, although body mass index (BMI) is widely used in clinical and epidemiological settings due to the simplicity of its calculation and interpretation—making it a popular tool for informing public health policies and interventions—its applicability in older populations is limited. BMI does not distinguish between fat mass and lean mass and may therefore mask sarcopenia and qualitative alterations in body composition [16,17]. Body cell mass represents the metabolically active component of fat-free mass, including muscle tissue and organ mass, which are directly involved in energy expenditure and functional capacity. Unlike BMI, BCMI reflects qualitative alterations in body composition that may precede weight loss and therefore may provide earlier signals of vulnerability in aging populations.

Consequently, frailty and malnutrition are increasingly understood as conditions requiring multidimensional assessment approaches that extend beyond traditional anthropometric indices. Despite growing international interest in this field, evidence from Albania remains scarce. This lack of country-specific data limits the development of targeted prevention and intervention strategies tailored to the local healthcare context.

Therefore, the objective of the present study was to improve the assessment of the health status of Albanian older adults, both community-dwelling and residing in long-term care facilities, by addressing both functional and nutritional components, using multidimensional questionnaires tailored to the physiology of aging and specific tools for the evaluation of body composition. For this reason, we decided to combine the use of the MNA, BIA, and BCMI, as this approach is particularly important in elderly Albanians, since single tools often fail to fully capture their complex nutritional status. The MNA may miss hidden malnutrition, while BIA alone lacks clinical context; BCMI complements BIA by reflecting cellular health. In this population, individuals can appear normal based on BMI or on the MNA but show abnormalities in body composition (e.g., low muscle mass or dehydration), or vice versa. This mismatch means that relying on one method can lead to under- or overestimation of risk. Therefore, a multi-parameter approach is necessary to detect conditions like sarcopenia, cachexia, or sarcopenic obesity, providing a more accurate and comprehensive assessment. Importantly, this approach also has international relevance. Although it is particularly valuable in transitional settings like Albania, similar discrepancies between clinical screening and body composition are increasingly recognized worldwide, especially in aging populations. As a result, combining the MNA, BIA, and BCMI might supports a more precise, multidimensional evaluation of nutritional status that can improve diagnosis, risk stratification, and management of malnutrition in elderly individuals across diverse healthcare contexts.

In addition, the study aimed to enhance the availability and quality of data on nutritional parameters beyond conventional anthropometric measures and to examine their associations with nutritional and functional risks in older adults.

## 2. Materials and Methods

### 2.1. Study Design and Sample

This cross-sectional observational study was conducted in Albania between February and July 2025 and included adults aged 65 years and older, both institutionalized (residing in long-term care facilities) and community-dwelling individuals. Participation was voluntary. Participants were recruited from three locations: Shkodër, Elbasan, and Tirana.

In Shkodër, participants included residents of long-term care facilities (five residential facilities: one public, three private, and one psychiatric supportive home) as well as community-dwelling older adults registered as patients at a primary healthcare center. In Elbasan, recruitment involved community-dwelling older adults registered at a city healthcare center. In Tirana, participants included residents of long-term care facilities and older adults attending a community center without residing there.

Inclusion criteria were age ≥ 65 years and the ability to participate in interviews and physical assessments. Exclusion criteria included acute illness at the time of evaluation, the presence of implanted electronic devices contraindicating bioelectrical impedance analysis, and clinical conditions associated with significant fluid imbalance.

A total of 292 older adults were invited to participate in the study. Of these, 194 were residents of residential care facilities in Shkodër, 15 were recruited from a residential care facility in Tirana, and 14 were from a community center in Tirana. In addition, 44 older adults presented on the announced days for questionnaires and measurements at a primary health center in Elbasan, and 25 at a primary health center in Shkodër.

Following data cleaning procedures, including the assessment of missing data, participants with incomplete questionnaire responses or measurements were excluded. The final sample consisted of 123 older adults. Specifically, thirty participants were excluded due to the presence of metal implants and lack of BIA measurement, despite having completed questionnaires. Nineteen participants were excluded due to cognitive or mental health issues resulting in incomplete questionnaires, and 22 participants were excluded due to missing data for various items.

### 2.2. Data Collection

Data were collected through face-to-face interviews conducted by trained fieldwork teams. All team members participated in a practical training session on standardized data collection procedures, delivered by members of the Albanian Society of Nutrition Science (ASNS). For institutionalized participants, evaluations were performed within residential facilities in Shkodër and Tirana, in dedicated rooms. For community-dwelling participants, assessments were carried out either at primary healthcare centers (in Shkodër and Elbasan) or community centers (Tirana). All BIA measurements were performed under standardized conditions. Whenever possible, participants were encouraged to refer to their medical documentation or ongoing treatments to improve the accuracy of the reported information.

Data were collected using standardized printed forms divided into three sections: (i) socio-demographic information, including sex, age, marital status, educational level, and place of residence (institutionalized or community-dwelling); (ii) anthropometric measurements; and (iii) nutritional and functional assessment questionnaires. All questionnaires were administered in Albanian. The instruments were translated by two independent bilingual Albanian–Italian academicians, and back-translation was performed to ensure accuracy and semantic equivalence.

### 2.3. Anthropometric Assessment, Body Composition Analysis and Malnutrition Classification

Body weight, height, and upper arm and calf circumferences were taken using calibrated portable equipment, with participants wearing light clothing and no shoes, in accordance with international criteria [18].

Body mass index (BMI) was calculated as weight (kg) divided by height squared (m^2^) and classified, according to the WHO Classification [19], as follows:

- Underweight: BMI ≤ 18.5 kg/m^2^;
- Normal weight: BMI 18.6–24.9 kg/m^2^;
- Overweight: BMI 25.0–29.9 kg/m^2^;
- Obesity: BMI ≥ 30.0 kg/m^2^.

Body composition was assessed using bioelectrical impedance analysis (BIA) with a phase-sensitive foot-to-hand impedance analyzer (BIA 101^®^, Akern, Florence, Italy) operating at a single frequency of 50 kHz. Raw bioelectrical parameter resistance (R), reactance (Xc), and phase angle (PhA) were obtained. Four low-intrinsic-impedance adhesive electrodes were placed on the left hand and foot. Measurements were performed with participants in the supine position on non-conductive surfaces, with the lower limbs abducted at 45° from the body’s midline and the upper limbs abducted at 30° from the trunk [20]. The whole procedure complied with international criteria [21]. Fat-free mass (FFM), fat mass (FM), total body water (TBW), extracellular water (ECW), and body cell mass (BCM) were estimated using predictive equations derived from raw electrical data. The body cell mass index (BCMI) was calculated as BCM (kg) divided by height squared (m^2^) and used as an indicator of metabolically active tissue.

Malnutrition was defined as:

- BMI ≤ 18.5 kg/m^2^ [19].
- Arm circumference < 21 [22].
- Calf circumference < 31 [22].
- Mini Nutritional Assessment (MNA) < 17 for malnutrition and MNA < 24 for risk of malnutrition [22,23]. The use of MNA < 24 as a combined outcome was intended to capture early nutritional impairment, including both established malnutrition and risk of malnutrition, which is particularly relevant in preventive geriatric contexts and can reflect any nutritional vulnerability, not just established malnutrition. Furthermore, combining these categories increases statistical power and avoids sparse data bias, and studies in the literature use MNA < 24 as a meaningful threshold. The approach reflects clinically relevant nutritional vulnerability, as all can benefit from early intervention. Additionally, due to the low prevalence of overt malnutrition, combining categories improved statistical power and is consistent with prior studies using MNA thresholds.
- BCMI < 8—International guidelines do not provide or endorse a universally validated BCMI cut-off; therefore, this threshold was adopted as an indicator of malnutrition, as previously applied in clinical BIA-based nutritional assessments [24,25,26,27].
- PhA < 5—In the literature, this mean value indicates compromised cellular health and nutritional status, and it has been associated with malnutrition, sarcopenia, and poorer outcomes in geriatric populations. Phase angle is derived from the relationship between resistance and reactance and reflects cell membrane integrity and body cell mass. Lower phase angle values are generally interpreted as indicative of reduced cellular integrity and altered hydration status [21,28,29,30].

### 2.4. Questionnaires

Nutritional status was evaluated using the full MNA, a validated screening tool for identifying malnutrition or risk of malnutrition in individuals aged 65 years and older across hospital, community, and long-term care settings [31]. The MNA consists of 18 items, including a screening section (maximum score: 14 points) and an assessment section (maximum score: 16 points). Participants were classified as:

- Well-nourished: 24–30 points;
- At risk of malnutrition: 17–23.5 points;
- Malnourished: <17 points.

Functional status was assessed using the Italian version (Grauer–Palombi Scale) [32] of the Grauer Geriatric Functional Evaluation Scale [33], a multidimensional instrument designed to evaluate physical, cognitive, and social functioning in older adults. The first section assesses physical and mental health status, including medical history, and assigns negative scores proportional to the degree of disability. The second section evaluates functional autonomy in daily activities, social and community support, and domestic and economic conditions, assigning positive scores that decrease with increasing disability. The Grauer Geriatric Functional Evaluation Scale was selected because it provides a multidimensional assessment of functional capacity, autonomy, and cognitive–behavioral components and has been previously applied in Southern European geriatric populations. Although internationally recognized tools such as the Fried Phenotype or the Clinical Frailty Scale are widely used, the Grauer scale was considered suitable for capturing broader functional impairment rather than exclusively physical frailty. Its use in this study reflects its multidimensional functional focus rather than its capacity to diagnose sarcopenia.

The final score, obtained by summing all individual items, allows classification into three categories:

- Category 1 (score < 20): Frail individuals, unable to live independently and requiring intensive home assistance or hospitalization.
- Category 2 (score 20–40): Pre-frail individuals, able to live at home with support services.
- Category 3 (score > 40): Robust individuals, able to live independently without significant external assistance.

### 2.5. Statistical Analysis

An anonymized database was created for statistical analysis. Data were analyzed using the Statistical Package for the Social Sciences (SPSS version 26.0; IBM Corp., Somers, NY, USA). Continuous variables were expressed as means and standard deviations (SDs), while categorical variables were presented as frequencies and percentages. Analyses were performed for the total sample and stratified by sex and age group (<75 and ≥75 years). Comparisons between groups were performed according to the distribution and size of the subgroups. Student’s *t*-test was used to assess differences in continuous variables between two groups when data were approximately normally distributed.

For comparisons between age groups (<75 years and ≥75 years), the Mann–Whitney U test was applied due to the relatively small subgroup sizes and the potential non-normal distribution of variables.

Categorical variables were compared using the chi-square test.

Questionnaire results were additionally analyzed according to place of residence (institutionalized vs. community-dwelling) and body-composition-based indices identifying malnutrition: phase angle (PhA < 5 vs. ≥5) and body cell mass index (BCMI < 8 vs. ≥8).

Odds ratios (ORs) with 95% confidence intervals (95% CIs) were calculated to examine the association between selected study variables (sex, living setting, BCMI, phase angle, and age) and questionnaire-based outcomes, including nutritional status assessed by the MNA and functional status assessed by the Grauer scale. Multivariable logistic regression models were then constructed to estimate adjusted odds ratios (AORs) and corresponding 95% CIs, with the aim of identifying independent determinants of frailty (defined as a Grauer score < 20) and malnutrition or risk of malnutrition (defined as an MNA score < 24). Given the presence of multiple independent variables, a forward stepwise regression approach was applied. Statistical significance was set at a two-sided *p*-value ≤ 0.05.

### 2.6. Ethical Consideration

Data collection commenced only after written informed consent was obtained from all participants prior to the study. Participation was voluntary, and confidentiality was ensured by assigning each participant a unique identification code. The study was conducted in accordance with the ethical principles of the Declaration of Helsinki of the World Medical Association (1975, as revised). This study was approved by the Ethics Committee of Catholic University of Our Lady of Good Counsel, Tirana (Approval No.: 033, date 6 February 2026).

## 3. Results

A total of 123 older adults participated in the study, of whom 56.9% were female. The mean age of the sample was 71.3 ± 7.4 years, with males being slightly older than females (73.1 ± 8.0 vs. 69.9 ± 6.6 years). A total of 54.5% were institutionalized, while 45.5% were community-dwelling.

Regarding socio-demographic characteristics, nearly half of the participants were widowed (42.3%), while 17.9% were single, 4.1% were divorced and approximately one third were married. In terms of living arrangements, 48.8% of the sample resided in residential care facilities, whereas 27.6% lived with family members and 17.1% with a partner; only a small proportion (6.5%) reported living alone. Educational attainment was generally low to moderate, with the majority of participants having completed secondary or high school education (approximately 70%), over 20% reporting only primary education, and a minority having attained university-level education (8.1%).

Anthropometric measurements indicated that mean arm and calf circumferences were within normal ranges in both sexes. The mean BMI of the overall sample was 27.5 ± 4.9 kg/m^2^, reflecting a high prevalence of excess body weight: 46.3% of participants were classified as overweight and 26.0% as obese. In contrast, only 24.4% of participants were of normal weight, and the prevalence of underweight was low (3.3%).

Body composition assessed by bioelectrical impedance analysis (BIA) showed marked sex-related differences. Males exhibited higher fat-free mass (FFM), total body water (TBW), extracellular water (ECW), body cell mass (BCM), and body cell mass index (BCMI) compared with females, consistent with greater lean tissue mass. Conversely, females showed higher fat mass (FM) percentage. Phase angle (PhA) values were comparable between sexes, suggesting similar overall cellular integrity at the group level.

Additional socio-demographic, anthropometric, and BIA-derived characteristics of the study population are presented in Table 1 for the total sample and stratified by sex.

Due to the relatively small subgroup sizes and the potential non-normal distribution of several variables, comparisons between age groups (<75 years and ≥75 years) were performed using the Mann–Whitney U test. Among women, statistically significant differences between age groups were observed for a limited number of body composition parameters. Arm circumference differed significantly between women aged < 75 years and those aged ≥ 75 years (*p* = 0.046). In addition, body cell mass (BCM) showed a significant difference between the two age groups (*p* = 0.018). A significant difference was also observed in fat mass percentage (FM%) (*p* = 0.033), suggesting age-related variations in body composition among female participants.

In contrast, no statistically significant differences were identified for body mass index (BMI) (*p* = 0.070), calf circumference (*p* = 0.058), resistance (*p* = 0.218), reactance (*p* = 0.262), phase angle (*p* = 0.059), total body water (TBW) (*p* = 0.103), extracellular water (ECW) (*p* = 0.467), or body cell mass index (BCMI) (*p* = 0.234).

Among men, comparisons between the two age groups did not reveal statistically significant differences for most anthropometric or bioelectrical impedance parameters. The only variable showing a statistically significant difference was reactance (*p* = 0.048).

Table 2 summarizes malnutrition prevalence according to different anthropometric, nutritional, and body composition indices, stratified by sex. Based on BMI classification, 3.3% of the overall sample was underweight, with a slightly higher prevalence among males than females. Similarly, the use of arm circumference (<21 cm) identified fewer than 5% of participants as malnourished; notably, all individuals classified as malnourished according to this indicator were female. In line with these findings, calf circumference (<31 cm) identified just over 7% of the total sample as malnourished, with a markedly higher prevalence among females compared with males (8 vs. 1 individuals). This sex-specific pattern was also observed when malnutrition was assessed using the MNA, which identified 2.4% of the sample as already malnourished, exclusively females. Conversely, when also considering people at risk of malnutrition (MNA < 24), this index was abnormal for slightly over 21% of the sample, being nearly double in women compared to men.

Generally, body-composition-based indices identified substantially higher rates of malnutrition. Using a BCMI cut-off < 8 kg/m^2^, 17.1% of participants were classified as malnourished, with a higher prevalence among females compared with males (18.6% vs. 15.1%). The highest prevalence of malnutrition was observed when phase angle (PhA < 5°) was applied, identifying 35.8% of the total sample as malnourished, again with a greater proportion among females than males.

Table 3 presents the results of the functional and nutritional assessments, as measured by the Grauer functional classification and the MNA, stratified by sex and living setting.

According to the functional questionnaire, 20.3% of the total sample was classified as frail, 13.0% as pre-frail, and 66.7% as robust. Frailty was more prevalent among females than males (25.7% vs. 13.2%), whereas males were more frequently classified as robust (73.6% vs. 61.4%). However, no statistically significant difference was observed in the mean total score between sexes.

In contrast, marked differences emerged when participants were stratified by living setting. Older adults residing in residential care facilities showed substantially higher proportions of frailty (31.3%) and pre-frailty (19.4%) compared with those living at home (7.1% and 5.4%, respectively). Consistently, the mean Grauer total score was significantly lower among institutionalized participants than among community-dwelling individuals (33.3 ± 27.7 vs. 55.4 ± 19.1; *p* < 0.001), indicating poorer functional status in residential settings.

Regarding nutritional status assessed by the MNA, 2.4% of the overall sample was classified as malnourished, all of whom were female, while 18.7% were identified as being at risk of malnutrition. Females exhibited a significantly lower mean MNA total score compared with males (24.8 ± 3.5 vs. 26.1 ± 2.3; *p* = 0.02). When stratified by living setting, no statistically significant differences were observed in mean MNA total scores between institutionalized and community-dwelling participants, although a higher proportion of individuals at risk of malnutrition was observed among those living at home (25.0% vs. 13.4%).

According to the Grauer questionnaire, overall, the most frequently self-reported morbidities among participants—showing a higher prevalence among community-dwelling older adults—were hypertension (48.0% in the total sample; 60.7% among those living at home vs. 37.3% among those residing in residential facilities), arthrosis (30.9%; 46.4% vs. 17.9%), dental problems (27.6%; 32.1% vs. 23.9%), cardiac diseases (26.8%; 37.5% vs. 17.9%), and diabetes (26.8%; 33.9% vs. 20.9%). Although less prevalent, this residence-related pattern was consistently observed across other morbidities.

**Figure 1:**
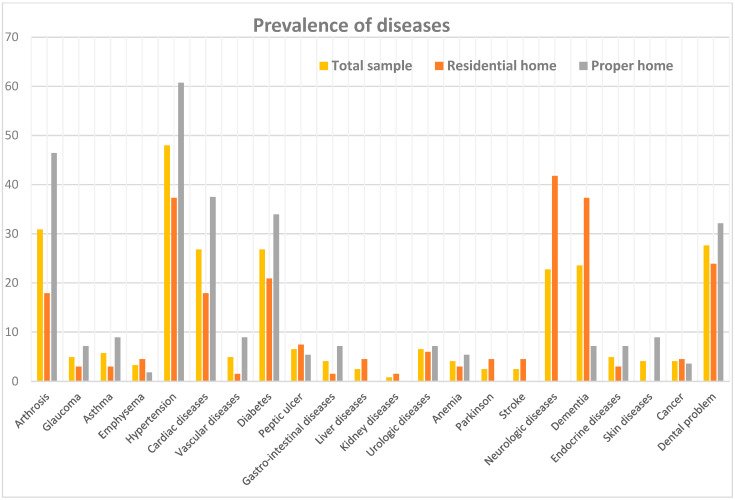
Self-reported health conditions, total and by living setting.

Conversely, conditions associated with greater functional impairment were predominantly reported among individuals living in residential care facilities. These included neurological diseases (22.8% overall; 0% among community-dwelling participants vs. 41.8% in residential care), dementia (23.6%; 7.1% vs. 37.3%), Parkinson’s disease (2.4%; 0% vs. 4.5%), and stroke (2.4%; 0% vs. 4.5%) (Figure 1).

**Figure A1:**
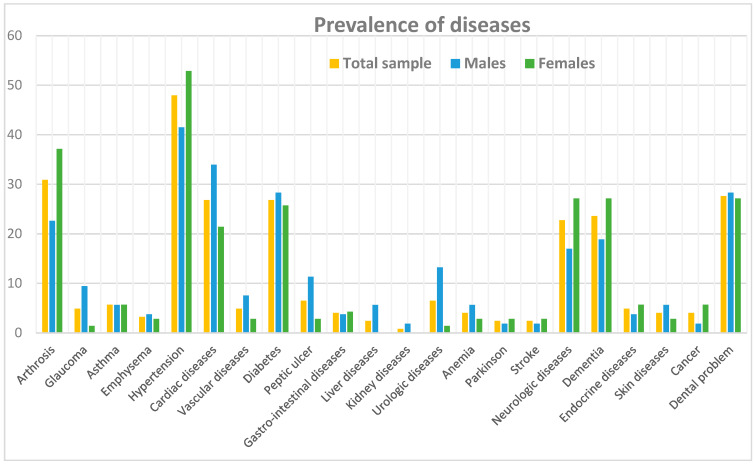
Self-reported health conditions by gender.

When stratified by sex, females showed a higher prevalence of hypertension, arthrosis, neurological diseases, dementia, and cancers, whereas males more frequently reported cardiac diseases, glaucoma, vascular diseases, kidney and liver disorders, urological diseases, and peptic ulcer disease. No substantial sex-related differences were observed for the remaining morbidities (Appendix A, Figure A1).

Table 4 reports questionnaire outcomes stratified by body-composition-derived malnutrition indices. When participants were stratified according to phase angle, no statistically significant differences were observed in the Grauer total score. Similarly, the mean MNA total scores did not differ significantly between the two PhA groups, despite a higher prevalence of malnutrition (4.5% vs. 1.3%) and risk of malnutrition (25.0% vs. 15.2%) when PhA < 5°.

In contrast, marked differences emerged when participants were split by the BCMI cut-off. Individuals with BCMI < 8 kg/m^2^ exhibited substantially poorer functional status, as reflected by a significantly lower Grauer total score compared with those with BCMI ≥ 8 kg/m^2^ (27.0 ± 34.5 vs. 46.8 ± 23.3; *p* = 0.002). Consistently, frailty was markedly more prevalent in the low-BCMI group (47.6%) than in participants with BCMI ≥ 8 kg/m^2^ (14.7%), while robust status was substantially less frequent (38.1% vs. 72.5%).

Similarly, nutritional status assessed by the MNA differed significantly according to BCMI classification. Participants with BCMI < 8 kg/m^2^ showed a significantly lower mean MNA total score (22.9 ± 3.9) compared with those with BCMI ≥ 8 kg/m^2^ (25.8 ± 2.7; *p* < 0.001), along with a higher prevalence of malnutrition (9.5% vs. 1.0%) and risk of malnutrition (38.1% vs. 14.7%).

Table 5 examines the main factors that, based on the study findings, may be associated with malnutrition and risk of malnutrition (defined as an MNA score < 24) and frailty (defined as a Grauer score < 20). The variables included both socio-demographic characteristics and body composition parameters. In univariate analyses, none of the socio-demographic variables showed a statistically significant association with an increased risk of frailty or malnutrition, whereas BCMI and institutionalization were independently associated with an increased risk of frailty. Although BCMI < 8 kg/m^2^ was included among descriptive malnutrition indicators, it was not used as an outcome variable in regression models. Instead, BCMI was examined as an independently associated factor of nutritional impairment defined by MNA < 24. This analytical approach was adopted to evaluate whether reduced metabolically active tissue is associated with questionnaire-based nutritional risk.

BCMI was also associated with poorer nutritional status, while PhA demonstrated weaker discriminatory capacity and did not reach statistical significance in this sample.

After adjustment for potential confounders, multivariable logistic regression analysis confirmed that low BCMI and residence in a long-term care facility were the factors with the strongest independent associations with frailty (AOR 5.02, 95% CI 1.69–14.87, *p* = 0.004 and AOR 5.71, 95% CI 1.76–18.54, *p* = 0.004, respectively). In addition, low BCMI was confirmed as the only variable associated with risk of malnutrition, confirming its role as a proxy indicator of impaired nutritional status (AOR 4.88, 95% CI 1.78–13.40, *p* = 0.002).

## 4. Discussion

In light of the increasing life expectancy and the growing proportion of older adults resulting from the global demographic transition, a multidimensional assessment of older adults is essential to better capture their overall health status and to support the early identification of functional and nutritional impairment before the onset of overt malnutrition and frailty. This approach is increasingly recognized as a public health priority in aging societies [34,35]. Although conducted in Albania, the multidimensional framework adopted in this study may be applicable to other countries experiencing rapid demographic aging and structural changes in long-term care systems, particularly in Eastern and South-Eastern Europe.

Frailty is increasingly recognized as a multidimensional condition encompassing nutritional, functional, cognitive, and clinical domains. The relatively young mean age of institutionalized participants may reflect the fact that admission to long-term care facilities in Albania is frequently driven by different comorbidities or lack of family support rather than advanced chronological age alone. In addition, it may have particular relevance from a prevention perspective, as understanding the age profile of the elderly population could help inform strategies aimed at the early identification and prevention of potential health conditions. From this perspective, comprehensive geriatric assessment—integrating functional performance measures, validated nutritional screening tools, and systematic evaluation of morbidities—allows for a more accurate characterization of frailty and its underlying correlated variables [36].

In the present cross-sectional analysis, institutionalization was associated with a higher burden of frailty-related characteristics; however, this observation should be interpreted as a descriptive association and does not imply a causal relationship between living setting and frailty status. Older adults residing in long-term care facilities often present with a higher burden of morbidities, reduced levels of physical activity, and a greater prevalence of malnutrition compared with community-dwelling peers, which may contribute to unfavorable changes in body composition and functional decline [37].

For this reason, the evaluation of older individuals should incorporate sustainable, multidimensional tools capable of addressing the various domains of daily life, including social circumstances, living conditions, functional capacity, and the presence of associated chronic diseases [38].

Our findings reveal a high prevalence of frailty, exceeding 20% of the overall sample, with a greater burden observed among women and older adults residing in long-term care facilities. When the living setting was considered, the prevalence of frailty among community-dwelling older adults was substantially below 10%, a finding consistent with results reported in older populations in Italy [32] as well as in other vulnerable populations studied in different contexts [39].

In our sample, especially among institutionalized individuals, a high prevalence of chronic diseases was observed, with findings for hypertension, diabetes, and cardiac diseases comparable to those reported by Ylli et al. [3].

With specific regard to nutritional assessment, although BMI is widely used in both clinical and epidemiological settings, its applicability in older populations is limited [40]. BMI does not differentiate between fat mass and lean mass and may therefore mask sarcopenia and qualitative alterations in body composition, particularly in the presence of overweight or obesity [16,17], especially in geriatric and institutionalized populations [9].

In contrast, BCM represents the metabolically active component of fat-free mass and includes tissues responsible for energy expenditure, protein turnover, and physical performance. For this reason, it has been proposed as one of the most informative indicators of nutritional status. BCM can be assessed using BIA, a portable, non-invasive, and low-cost method that allows for the evaluation of body cell mass and hydration status [41].

The BCMI, which normalizes BCM for height, has been suggested as a more sensitive indicator of nutritional and functional status in older adults compared with BMI alone. Lower BCMI values have consistently been associated with malnutrition, impaired physical performance, and increased frailty in several clinical contexts, particularly among individuals with a high burden of chronic disease. However, the clinical interpretation of BCMI remains heterogeneous across studies, and currently available evidence is largely derived from specific populations, limiting direct comparability with the international literature and broader generalizability. Consequently, BCMI should be interpreted cautiously and cannot be considered a central or standalone marker of nutritional status without further external validation in diverse populations [17].

Despite growing international interest in the complex interplay between body composition, nutritional status, multimorbidity, and frailty [35], evidence from Albania remains scarce, limiting the development of targeted prevention and intervention strategies tailored to the local context. To the best of our knowledge, this pilot study represents one of the first multidimensional investigations adopting a multidisciplinary approach to frailty and nutritional assessment among older adults in Albania. First, our results highlight a marked sex-specific pattern in age-related changes in body composition. Females exhibited more pronounced declines in parameters associated with metabolically active tissue and cellular integrity, whereas males showed relatively preserved anthropometric and BIA-derived measures across age groups. These sex-related differences in body composition may help explain the higher vulnerability to frailty observed among older women, particularly in advanced age, as reductions in metabolically active tissue and cellular integrity are known to adversely affect functional reserve and resilience [42]. However, these differences should be interpreted as descriptive patterns rather than causal mechanisms underlying frailty. In addition, functional impairment and frailty were strongly associated with institutionalization, underscoring the vulnerability of older adults residing in long-term care facilities, although no directional or causal inference can be established due to the study design. Overall, these findings emphasize the added value of BIA-derived parameters in characterizing body composition beyond conventional anthropometric indices and reinforce the need for multidimensional assessment approaches in geriatric populations.

An important finding of this study is the discrepancy between the relatively low prevalence of malnutrition identified by the MNA and the substantially higher proportion of individuals classified as at risk according to BCMI and PhA. This difference may be explained by the distinct constructs captured by these tools: while the MNA reflects a multidimensional evaluation including dietary intake, weight loss, and functional and cognitive aspects, BCMI and PhA are primarily indicators of body composition and cellular integrity. Therefore, BIA-derived parameters may detect early or subclinical alterations in metabolically active tissue that are not yet captured by questionnaire-based tools. Alternatively, this discrepancy may reflect differences in sensitivity and specificity, as well as the absence of standardized cut-off values for BCMI and PhA, potentially leading to an overestimation of malnutrition risk when using these indicators alone.

Furthermore, our results indicate that body-composition-derived parameters (i.e., BCMI and PhA) identified a substantially higher proportion of individuals at risk of malnutrition compared with body mass index and other conventional anthropometric measures, such as arm and calf circumferences. Additionally, among all variables considered in the present analysis, BCMI was found to be associated with both functional and nutritional assessment outcomes. Specifically, lower BCMI values were significantly associated with poorer functional status as assessed by the Grauer scale, indicating a higher risk of frailty, as well as with lower MNA scores, reflecting an increased risk of malnutrition. However, these associations should be interpreted with caution, as they do not imply predictive or causal relationships and may be influenced by unmeasured confounding factors. In contrast, other socio-demographic, anthropometric, and bioelectrical parameters showed weaker or non-significant associations with questionnaire-based outcomes. Overall, while these findings suggest that BCMI may provide additional information on body composition in older adults—in line with previous evidence reporting low BCMI values as indicators of malnutrition and increased nutritional risk across different clinical populations [17]—its role should be considered complementary rather than definitive within comprehensive geriatric assessment. Further longitudinal and validation studies are needed to clarify its clinical utility and to establish robust and widely applicable reference thresholds.

### Limitations

Several limitations should be acknowledged when interpreting the findings of this study. First, the relatively small sample size of this pilot study represents one of its main limitations and may reduce the statistical power and generalizability of the results. Second, the cross-sectional design does not allow causal inferences to be drawn. The observed associations should therefore be interpreted as exploratory and hypothesis-generating rather than confirmatory.

In addition, all socio-demographic information and data on health conditions and morbidities were self-reported. Consequently, the assessment of multimorbidity may have been subject to recall bias and may have led to an overestimation of the prevalence of certain conditions among participants.

Another limitation concerns the use of assessment instruments that have not been formally validated in the Albanian context. Although the Grauer scale was translated using a forward–backward translation procedure performed by two independent bilingual academics, the lack of local validation may affect the reliability and cultural appropriateness of the measurements.

With specific regard to body composition, bioelectrical impedance analysis (BIA) may be subject to systematic measurement errors, particularly in institutionalized individuals, where hydration status, mobility limitations, and clinical conditions may influence the accuracy of estimates. Furthermore, there are no internationally standardized reference values or universally accepted cut-off points for either BCMI or PhA, limiting their comparability across different populations and clinical settings [43]. Most proposed thresholds derive from single-center studies conducted in specific patient groups, thereby restricting their applicability to broader and more heterogeneous cohorts [17]. Therefore, the cut-off values applied in the present study should be interpreted with caution and should not be considered universally applicable across all populations. The threshold of BCMI < 8 kg/m^2^ used in this study should be interpreted as an exploratory and promising indicator rather than a diagnostic criterion, as it is derived from previous clinical applications but has not been universally validated in older populations. Therefore, caution is warranted when generalizing these findings.

Furthermore, the use of MNA < 24 as a combined outcome was intended to capture early nutritional impairment; however, the results depend strongly on the arbitrary choice of threshold values.

The higher prevalence of self-reported chronic conditions among community-dwelling individuals may reflect greater awareness of diagnoses, as well as potential underdiagnosis and underreporting in institutional settings.

Finally, participants were recruited using a convenience sampling strategy across selected facilities and community settings; therefore, the sample cannot be considered nationally representative of the older Albanian population. Taken together, these methodological limitations may limit the generalizability of the findings. 

## 5. Conclusions

The present exploratory cross-sectional study provides preliminary insight into the assessment of health and nutritional status among older adults living in Albania, including both institutionalized and community-dwelling individuals. The findings suggest the potential relevance of integrating body composition assessment into routine geriatric evaluation, particularly for the early detection of frailty and malnutrition-related conditions.

A key strength of this study is its comprehensive evaluation framework, which integrates functional assessment, nutritional screening, morbidity burden, and body composition parameters. This multidimensional approach allows for a broad characterization of frailty-related profiles across different care settings, without implying causal relationships among the observed variables. Our findings also highlight the inclusion of additional parameters for malnutrition screening in the elderly population for public health preventive purposes. In particular, BCMI emerges as a potentially useful and promising body-composition-derived indicator for the assessment of nutritional status, as well as for the detection of conditions related to frailty across diverse populations and care settings. However, these associations should be interpreted cautiously, given the cross-sectional nature of the study. Finally, considering the limited availability of data on aging and elderly care in Albania, this study should be regarded as an initial contribution aimed at generating baseline evidence. While the findings may offer insights relevant to other countries undergoing similar demographic and socioeconomic transitions, further longitudinal and confirmatory research is needed to better understand the observed relationships [44,45].

## Figures and Tables

**Table 1:** Socio-demographic characteristics and anthropometric characteristics by total and by gender.

Variables	Total n 123 (100%)	Females n 70 (56.9%)	Males n 53 (43.1%)
Mean age ± SD (years)	71.3 ± 7.4	69.9 ± 6.6	73.1 ± 8.0
Education			
None	4 (3.3)	4 (5.7)	0 (0)
Primary	25 (20.3)	14 (20.0)	11 (20.8)
Secondary	49 (39.8)	26 (37.1)	23 (43.4)
High school	35 (28.5)	20 (28.6)	15 (28.3)
University	10 (8.1)	6 (8.6)	4 (7.5)
Marital status			
Single	22 (17.9)	11 (15.7)	11 (20.8)
Married	44 (35.8)	21 (30.0)	23 (43.4)
Divorced	5 (4.1)	3 (4.3)	2 (3.8)
Widow	52 (42.3)	34 (48.6)	18 (34)
Living conditions			
Alone	8 (6.5)	6 (8.6)	2 (3.8)
With partners	21 (17.1)	11 (15.7)	10 (18.9)
With family members	34 (27.6)	15 (21.4)	19 (35.9)
In residential houses	60 (48.8)	38 (54.3)	22 (41.5)
Anthropometric measurements			
Weight ± SD (kg)	73.8 ± 14.0	71.6 ± 15.3	76.8 ± 11.6
Height ± SD (cm)	164.1 ± 9.2	159.6 ± 7.8	170.1 ± 7.3
Body mass index ± SD (kg/m^2^)	27.5 ± 4.9	28.1 ± 5.5	26.7 ± 3.9
Arm circumference ± SD (cm)	28.4 ± 3.9	29.1 ± 4.5	27.4 ± 2.7
Calf circumference ± SD (cm)	35.6 ± 4.2	36.1 ± 4.6	34.8 ± 3.2
Nutritional status, Body Mass Index			
Underweight	4 (3.3)	2 (2.9)	2 (3.8)
Normal weight	30 (24.4)	14 (20.0)	16 (30.2)
Overweight	57 (46.3)	32 (45.7)	25 (47.2)
Obesity	32 (26.0)	22 (31.4)	10 (18.9)
BIA measurements			
Resistance ± SD ( 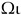 )	529.6 ± 79.3	546.09 ± 86.1	507.8 ± 63.6
Reactance ± SD ( 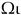 )	49.5 ± 9.7	50.7 ± 10.1	47.9 ± 9.0
Phase Angle ± SD (°)	5.3 ± 0.8	5.3 ± 0.8	5.4 ± 0.9
Fat Mass ± SD (kg)	51.1 ± 8.2	46.6 ± 6.6	57.2 ± 6.1
Fat Free Mass± SD (%)	69.9 ± 8.9	66.3 ± 8.3	74.8 ± 7.3
Total Body Water ± SD (L)	37.7 ± 6.2	34.4 ± 5.1	42.1 ± 4.7
Total Body Water ± SD (%)	51.8 ± 6.8	49.0 ± 6.1	55.4 ± 5.8
Extra Cellular Water ± SD (L)	18.5 ± 3.2	16.9 ± 2.5	20.6 ± 2.9
Body Cell Mass ± SD (kg)	25.6 ± 5.2	23.2 ± 4.5	28.8 ± 4.3
Fat Mass ± SD (%)	30.0 ± 8.9	33.7 ± 8.3	25.1 ± 7.3
Body Cell Mass Index ± SD (kg/m^2^)	9.5 ± 1.5	9.2 ± 1.5	9.9 ± 1.5

**Table 2:** Malnutrition classification by different indices and by gender.

Malnutrition Classification	Total n 123 (100%)	Females n 70 (56.9%)	Males n 53 (43.1%)
Body mass index < 18.5	4 (3.3)	2 (2.9)	2 (3.8)
Arm circumference < 21	6 (4.9)	4 (5.7)	2 (3.8)
Calf circumference < 31	9 (7.3)	8 (11.4)	1 (1.9)
Mini Nutritional Assessment < 17	3 (2.4)	3 (4.9)	0 (0.0)
Mini Nutritional Assessment < 24	26 (21.1)	17 (24.3)	9 (16.9)
Body Cell Mass Index < 8	21 (17.1)	13 (18.6)	8 (15.1)
Phase Angle < 5	44 (35.8)	27 (38.6)	17 (32.1)

**Table 3:** Functional and nutritional status based on questionnaire results by sex and living setting.

Questionnaire Results	Total n 123 (100%)	Females n 70 (56.9%)	Males n 53 (43.1%)	*p*-Value	Institutionalized n 67 (54.5%)	Community-Dwelling n 56 (45.5%)	*p*-Value
Grauer classification							
Frail	25 (20.3)	18 (25.7)	7 (13.2)		21 (31.3)	4 (7.1)	
Pre-frail	16 (13.0)	9 (12.9)	7 (13.2)		13 (19.4)	3 (5.4)	
Robust	82 (66.7)	43 (61.4)	39 (73.6)		33 (49.3)	49 (87.5)	
Total score, mean ± SD	43.4 ±26.5	41.4 ± 29.3	46.1 ± 22.1	NS	33.3 ± 27.7	55.4 ± 19.1	>0.001
Mini Nutritional Assessment classification							
Malnutrition	3 (2.4)	3 (4.3)	0 (0.0)		3 (4.5)	0 (0.0)	
At risk of malnutrition	23 (18.7)	14 (20.0)	9 (17.0)		9 (13.4)	14 (25.0)	
Good nutritional status	97 (78.9)	53 (75.7)	44 (83.0)		55 (82.1)	42 (75.0)	
Mini Nutritional Assessment total score, mean ± SD	25.3 ± 3.1	24.8 ± 3.5	26.1 ± 2.3	0.02	25.4 ± 3.4	25.2 ± 2.8	NS

**Table 4:** Functional and nutritional questionnaire outcomes according to body-composition-derived malnutrition indices.

Questionnaire Results	Total n 123 (100%)	Phase Angle < 5°	PhA ≥ 5°	*p*-Value	Body Cell Mass Index < 8 kg/m^2^	BCMI ≥ 8 kg/m^2^	*p*-Value
Grauer classification							
Frail	25 (20.3)	11 (25.0)	14 (17.7)		10 (47.6)	15 (14.7)	
Pre-frail	16 (13.0)	3 (6.8)	13 (16.5)		3 (14.3)	13 (12.7)	
Robust	82 (66.7)	30 (68.2)	52 (65.8)		8 (38.1)	74 (72.5)	
Total score, mean ± SD	43.4 ± 26.5	44.3 ± 30.1	42.9 ± 24.4	NS	27.0 ± 34.5	46.8 ± 23.3	0.002
Mini Nutritional Assessment classification							
Malnutrition	3 (2.4)	2 (4.5)	1 (1.3)		2 (9.5)	1 (1.0)	
At risk of malnutrition	23 (18.7)	11 (25.0)	12 (15.2)		8 (38.1)	15 (14.7)	
Good nutritional status	97 (78.9)	31 (70.5)	66 (83.5)		11 (52.4)	86 (84.3)	
Mini Nutritional Assessment total score, mean ± SD	25.3 ± 3.1	24.8 ± 3.5	25.6 ± 2.8	NS	22.9 ± 3.9	25.8 ± 2.7	<0.001

**Table 5:** Univariate and multivariate logistic regression analyses for factors associated with nutrition impairment and frailty.

Variable	Univariate Analysis		Multivariate Analysis	
	Odds Ratio (95% CI)	*p*-Value	Adjusted Odds Ratio (95% CI)	*p*-Value
Frailty (Grauer < 20)				
Gender (female)	2.27 (0.87–5.93)	NS		
Age (over 75 years)	0.74 (0.29–1.88)	NS		
Living setting (institutionalized)	5.93 (1.89–18.56)	0.001	5.71 (1.76–18.54)	0.004
Body Cell Mass Index (<8 kg/m^2^)	5.27 (1.90–14.57)	0.002	5.02 (1.69–14.87)	0.004
Phase Angle (<5°)	1.54 (0.63–3.78)	NS		
Malnutrition (Mini Nutritional Assessment < 24)				
Gender (female)	1–56 (0.63–3.86)	NS		
Age (over 75 years)	0.52 (0.21–1.28)	NS		
Living setting (Residential home)	0.65 (0.27–1.56)	NS		
Body Cell Mass Index (<8 kg/m^2^)	4.88 (1.78–13.40)	0.003	4.88 (1.78–13.40)	0.002
Phase Angle (<5°)	2.12 (0.88–5.12)	NS		

## Data Availability

Data will be made available upon reasonable request. The data presented in this study are available on request from the corresponding author due to ethical restrictions.

## Abbreviations

The following abbreviations are used in this manuscript:

ASNS	Albanian Society of Nutrition Science
BCM	Body Cell Mass
BCMI	Body Cell Mass Index
BIA	Bioelectrical Impedance Analysis
BMI	Body Mass Index
ECW	Extracellular Water
FFM	Fat-Free Mass
FM	Free Mass
MNA	Mini Nutritional Assessment
PhA	Phase Angle
R	Resistance
TBW	Total Body Water
WHO	World Health Organization
Xc	Reactance
